# Saliva-derived microcosm biofilms grown on different oral surfaces in vitro

**DOI:** 10.1038/s41522-021-00246-z

**Published:** 2021-09-09

**Authors:** Xiaolan Li, Lin Shang, Bernd W. Brandt, Mark J. Buijs, Sanne Roffel, Cor van Loveren, Wim Crielaard, Susan Gibbs, Dong Mei Deng

**Affiliations:** 1 Department of Operative Dentistry and Endodontics, Guanghua School of Stomatology & Hospital of Stomatology, Guangdong Province Key Laboratory of Stomatology, Sun Yat-sen University, Guangzhou, China; 2 Department of Preventive Dentistry, Academic Centre for Dentistry Amsterdam (ACTA), University of Amsterdam and Vrije Universiteit Amsterdam, Amsterdam, The Netherlands; 3 Department of Oral Cell Biology, Academic Centre for Dentistry Amsterdam (ACTA), University of Amsterdam and Vrije Universiteit Amsterdam, Amsterdam, The Netherlands; 4 Department of Molecular Cell Biology and Immunology, Amsterdam UMC, Vrije Universiteit Amsterdam, Amsterdam, The Netherlands

**Keywords:** Symbiosis, Microbial ecology

## Abstract

The microbial composition of a specific oral niche could be influenced by initial bacterial adherence, nutrient and physiological property of the local surface. To investigate the influence of nutrient and surface properties on microbial composition, saliva-derived biofilms were grown in agar on three substrata: Reconstructed Human Gingiva (RHG), a hydroxyapatite (HAP) surface, and a titanium (TI) surface. Agar was mixed with either Brain Heart Infusion (BHI) or Thompson (TP) medium. After 1, 3, or 5 days, biofilm viability (by colony forming units) and microbiome profiles (by 16 S rDNA amplicon sequencing) were determined. On RHG, biofilm viability and composition were similar between BHI and TP. However, on the abiotic substrata, biofilm properties greatly depended on the type of medium and substratum. In BHI, the viability of HAP-biofilm first decreased and then increased, whereas that of TI-biofilm decreased in time until a 6-log reduction. In TP, either no or a 2-log reduction in viability was observed for HAP- or TI-biofilms respectively. Furthermore, different bacterial genera (or higher level) were differentially abundant in the biofilms on 3 substrata: *Haemophilus* and *Porphyromonas* for RHG; *Bacilli* for HAP and *Prevotella* for TI. In conclusion, RHG, the biotic substratum, is able to support a highly viable and diverse microbiome. In contrast, the viability and diversity of the biofilms on the abiotic substrata were influenced by the substrata type, pH of the environment and the richness of the growth media. These results suggest that the host (oral mucosa) plays a vital role in the oral ecology.

## Introduction

The human body is colonized by millions of microbes. These microbes form diverse and dynamic microbial communities distributed over various body habitats, including the skin, gut and oral cavity. High-throughput sequencing technologies revealed that the microbial community of each niche has its own distinct biodiversity features^1–4^. Niche-specific structure maintains the ecological stability of the microbiome community and ensures a healthy balance between host and microbes. The loss of this structure can eventually lead to diseases^5,6^. Hence, identification of major factors influencing the niche-specificity is critical for maintaining a healthy microbial ecology.

The oral cavity is a complex habitat, comprising of diverse sites characterized by different anatomic structures and physiochemical factors^7^. These sites include natural substrata, such as hard (teeth) and soft (mucosa) tissues, and therapeutic substrata, such as dental implants and dentures, where various microbes can colonize and flourish. Oral microbiomes also show niche-specific structures: separate sites within the oral cavity, whilst being bathed in the same saliva, harbor considerably different microbial communities^2,8,9^. For example, the keratinized gingiva is mainly occupied by *Gemella haemolysans* and *Streptococcus mitis* group species, whereas the supragingival plaque on tooth surfaces is dominated by *Rothia, Streptococcus* and *Corynebacterium*^9,10^. Thus, Welch et al. proposed a “Site-Specialist Hypothesis”: an oral microbe can actively colonize its preferred site, grow and divide there. When outside its preferred site, it will be in much lower abundance and display altered metabolism, gene expression and spatial organization.

According to this hypothesis, the ability of a microbe to adhere to a surface, directly or indirectly, determines its site-specificity and thereby the local microbial community. Bacterial adherence is only the first step in biofilm development. Other factors during biofilm formation, e.g. nutrients supplied by saliva or local substrata, also affect the niche-specific microbiome structure. It was demonstrated that titanium released from dental implants led to the reduction of alpha-diversity and enrichment of *Veillonella* and *Neisseria* in the (subgingival) peri-implant microbiome^11^. However, an in vitro study investigated the effect of different abiotic substrata (glass versus hydroxyapatite discs) and two types of growth media on the composition of saliva-derived microcosm biofilms and revealed that the growth media, not the substrata, mainly affected the structure of the microbiome^12^. Therefore, the influence of nutrients, substrata and their interactions on the niche-specific microbiome structure warrants further investigation.

Our aim was to investigate the influence of nutrient and oral substrata on the community structure of a saliva-derived microcosm biofilm grown for 1, 3 and 5 days. Two biofilm growth media, BHI (brain heart infusion broth) and TP (Thompson medium^13^), were examined. BHI is a protein-rich medium. Multi-species oral biofilms have been successfully cultured in BHI, supplemented with menadione and hemin^14,15^. TP is a complex medium containing proteose peptone, mucins and serum, which supports nutrient requirements of subgingival biofilms^16,17^. Three substrata were used to support the growth of biofilms: reconstructed human gingiva (RHG; differentiated, stratified human gingival epithelium on a fibroblast populated collagen hydrogel), hydroxyapatite discs (HAP) and titanium discs (TI), representing 3 oral sites respectively: gingiva, tooth and dental implant. Previously, RHG has shown similar reactions to oral multi-species biofilms as native gingiva, such as the induction of defensive cytokines and antimicrobial peptides^18,19^. RHG represents a biotic substratum, whereas HAP and TI are abiotic substrata.

## Results

### Microcosm biofilm viability and RHG histology

**Fig. 1:**
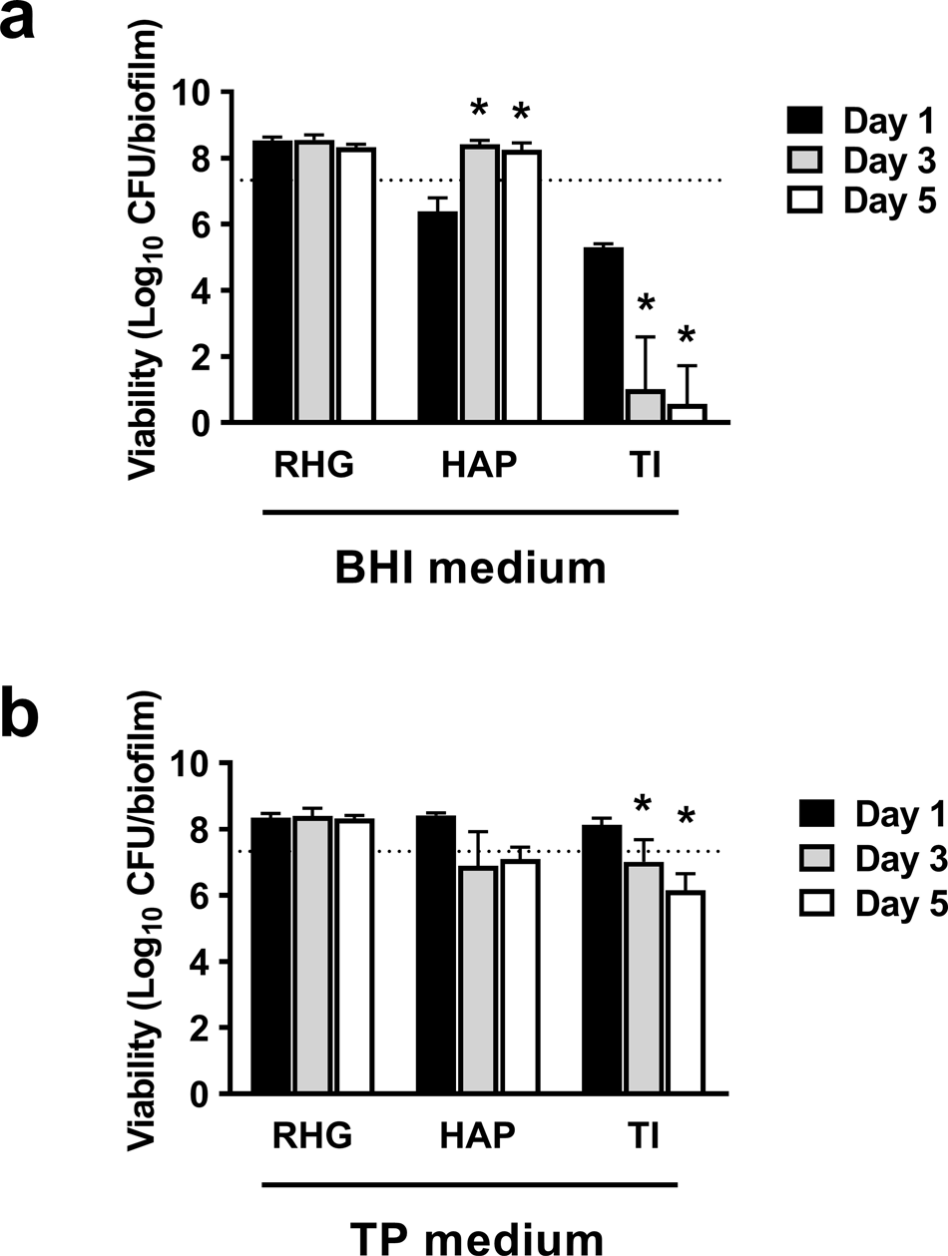
Viability of microcosm biofilms grown on different oral substrata. The saliva inoculum was mixed with 0.7% agar containing biofilm growth media—either BHI (**a**) or TP (**b**), and inoculated onto one of the substrata (RHG, HAP or TI). The biofilms were collected for viable cell counts on day 1, 3 and 5. RHG: reconstructed human gingiva; HAP: hydroxyapatite discs; TI: titanium discs. Data represent the mean ± standard deviation of three independent experiments. * represents statistically significant difference when compared to the day 1 biofilms (*p* < 0.05). The dotted line indicates the averaged viable cell counts of saliva inocula of all experiments.

Figure 1 shows differences in biofilm viability grown on 3 substrata in time in BHI or TP medium. With BHI, the biofilm viability was clearly determined by the substrata type. Viable counts of the biofilms grown on RHG increased from 7.3 ± 0.1 log_10_CFU/biofilm at inoculation (dotted line) to 8.5 ± 0.5 log_10_CFU/biofilm after 1 day and remained at this level until day 5. However, the viable counts of those on HAP decreased to 6.4 ± 0.1 log_10_CFU/biofilm after 1 day but increased to 8.4 ± 0.1 log_10_CFU/biofilm on day 3 and 5. The viable counts of those on titanium (TI), decreased over time and was below detection limit (2.5 log_10_CFU/biofilm) from day 3. In contrast, with TP, biofilm viability was much less affected by the type of substratum. On RHG and HAP, biofilm viable counts reached approximately 8.4 log_10_CFU/biofilm after 1 day and remained at this level until day 5. On TI, biofilm viable counts significantly decreased over time. However, the reduction was within 1.9 log_10_CFU/biofilm, much less than when biofilms were grown in BHI. The unexposed RHG showed a differentiated and stratified epithelium on a fibroblast-populated collagen hydrogel. Application of the agar, with or without microcosm biofilm, moderately disrupted the epithelium in localized places, irrespective of the growth media (Supplementary Fig. 1).

### Microbial profiles of microcosm biofilms on different substratum

**Fig. 2:**
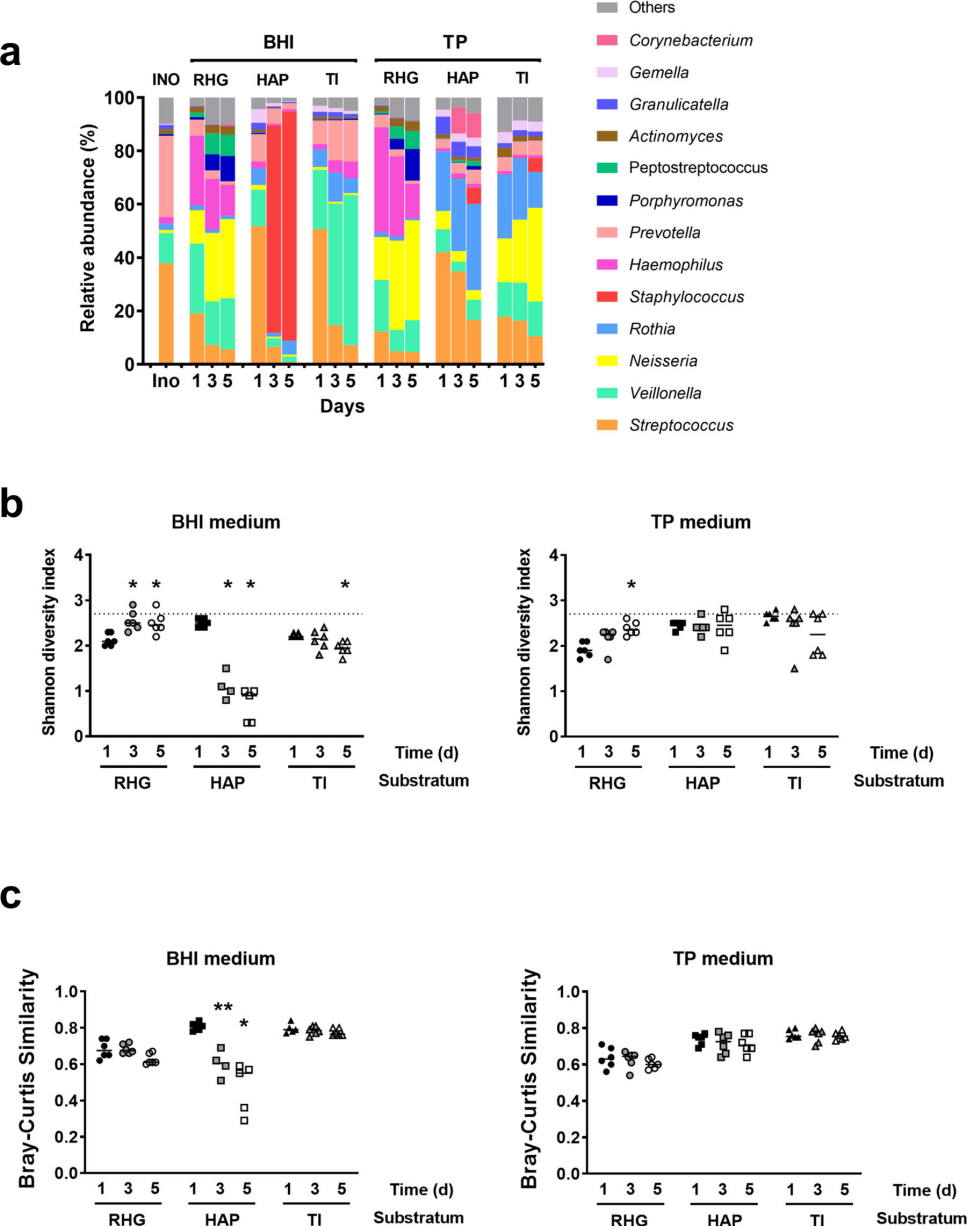
Microbial profiles of microcosm biofilms on each oral substratum. **a** Relative abundance of bacterial genera in microcosm biofilms. In total, 13 genera that are found at a relative abundance higher than 1% in any of the samples, were included and counted. The remaining genera are grouped as others. **b** The Shannon diversity index of microcosm biofilms grown in either BHI (left) or TP (right). The dotted line indicates the Shannon index of the saliva inoculum. * represents statistically significant difference when compared to the day 1 biofilms for each condition (*p* < 0.05). **c** Bray-Curtis similarity index between the saliva inoculum and the biofilms grown in either BHI (left) or TP (right) medium for 1, 3 and 5 days. * represents statistically significant difference when compared to the sample pair of day 1 biofilms vs. inoculum (*p* < 0.05). INO, saliva inoculum; RHG: Reconstructed Human Gingiva; HAP: hydroxyapatite discs; TI: titanium discs. TP: Thompson medium. Data represent the average of 3 independent experiments, each performed in replicates.

Figure 2a gives an overview of the relative abundance of the bacterial genera in different biofilms. Generally, the distribution pattern was independent of biofilm age per substratum and growth medium, with the exception of those grown on HAP in BHI medium: on day 1, this biofilm showed a diverse community, but on day 3 and 5, it was dominated by *Staphylococcus*. Indeed, analysis based on Shannon index, showed a significant reduction in biofilm α-diversity of these biofilms from day 1 to day 3 and 5 (Fig. 2b). However, no significant reduction in α-diversity was observed for HAP biofilms grown in TP medium. Interestingly, the Shannon indexes of RHG biofilms in both growth media were first lower than that of the saliva inoculum and then increased over time. The increases relative to day 1 were significant from day 3 (BHI) and on day 5 (BHI and TP).

Figure 2c demonstrates the Bray-Curtis (BC) similarity indexes between the saliva inoculum and microcosm biofilms. Two-way ANOVA analysis showed that the BC similarity indexes of RHG-biofilms were consistently lower than those of HAP-biofilms (in TP medium) and TI-biofilms (in BHI and TP media), irrespective of biofilm age. The differences were small but significant. Further one-way ANOVA analysis demonstrated that the BC indexes of HAP-biofilms in BHI medium reduced considerably with the increasing biofilm ages, indicating a clear shift in the microbial composition of these biofilms from the original saliva inoculum. However, the indexes of the rest biofilms remained stable over time.

### Ordination analysis of microcosm biofilms on different substratum

**Fig. 3:**
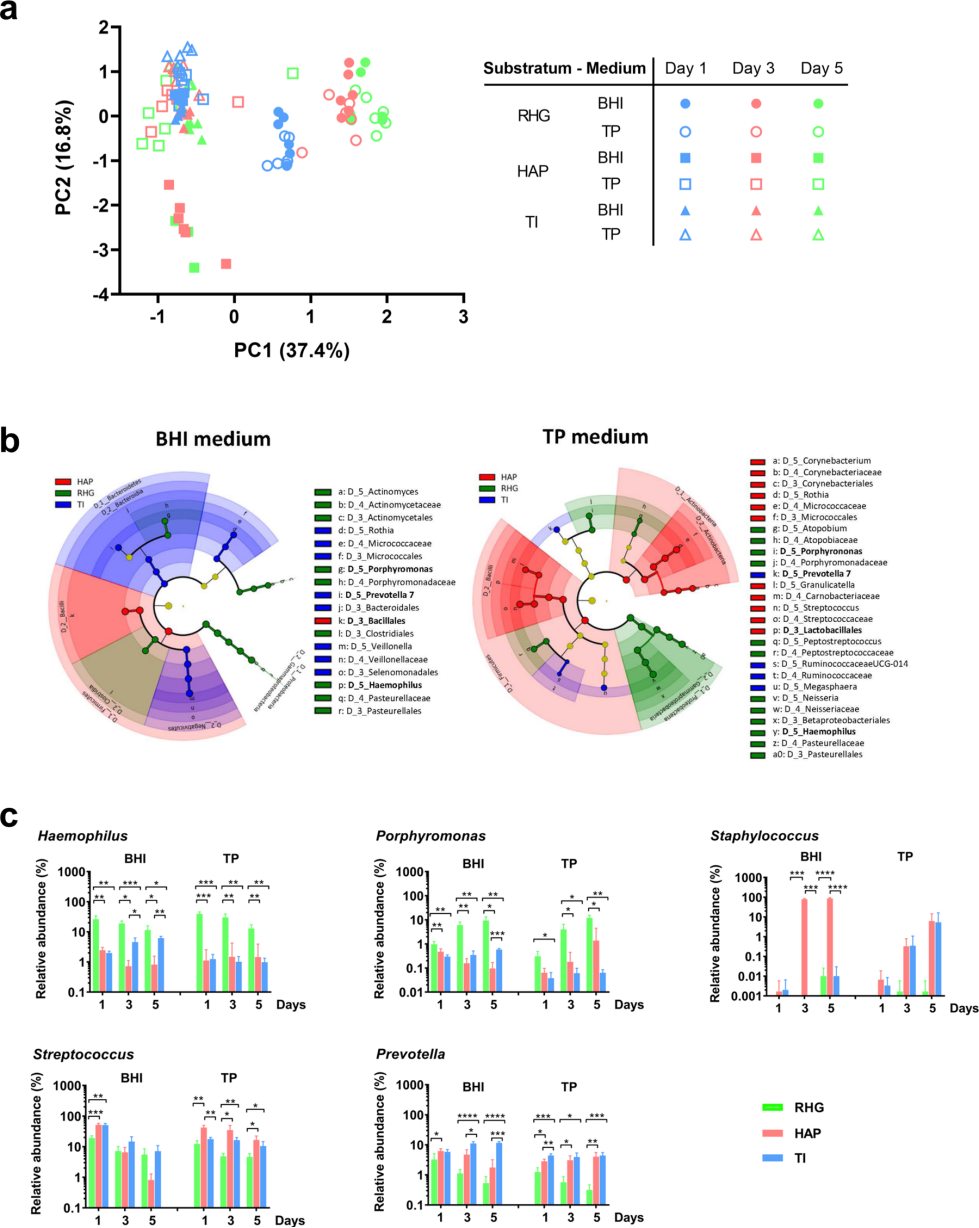
Ordination of microcosm biofilms grown under different conditions. **a** Principal component analysis (PCA) plot of microcosm biofilms grown in BHI or TP and on different substratum (RHG, HAP or TI) over time (day 1, 3 or 5). **b** Cladogram represents the differentially abundant orders, families and genera in the biofilms grown on the surfaces of RHG, HAP and TI when the growth medium was BHI or TP. The root denotes the domain bacteria. The taxonomic level of class is labeled, while order, family and genus are abbreviated. The color shade indicates the abundance of substratum-specific taxa. The size of each node represents their relative abundance. The cladogram was plotted based on the logarithmic LDA score calculated in LEfSe with an effect size cutoff of 4.0. The bacterial genera or higher level appeared in both BHI and TP medium on the same substratum are in bold. **c** The relative abundance (%) of individual bacterial genera in microcosm biofilms grown on the surfaces of RHG, HAP and TI. The substratum-specific individual bacterial genera (or higher level) were selected based on the results shown in the cladogram: *Haemophilus* and *Porphyromonas* for RHG-biofilms; the class *Bacilli* for HAP-biofilm and *Prevotella* for TI-biofilms. The relative abundances of *Staphylococcus* and *Streptococcus*, representatives of *Bacillales* and *Lactobacillales*, are shown. Data are shown as mean ± standard deviation. The * represents statistically significant difference (*p* < 0.05) compared using two-way ANOVA with Bonferroni’s multiple comparisons test in Prism 8 version 8.2.1. RHG: Reconstructed Human Gingiva; HAP: hydroxyapatite discs; TI: titanium discs. TP: Thompson medium. Data represent the average of 3 independent experiments, each performed in replicates.

In order to understand the growth media- and substrata-dependent shifts in biofilm compositions over time, principal component analysis (PCA) and LEfSe were performed. The PCA plot identified four major clusters (Fig. 3a). HAP- and TI-biofilms were clustered to the left side of *PC1*-axis, whereas RHG-biofilms were clustered to the right side. Biofilms on the left side of *PC1*-axis were separated with the majority clustering at the upper side of the *PC2*-axis and HAP-biofilms of day 3 and 5 at the lower side of the *PC2-*axis. For RHG-biofilms, a clear shift in composition was observed between day 1 and day 3/5. The type of growth media seemed to have little influence on the composition of RHG-biofilms. The last observation from PCA plot is confirmed by the Two-way PERMANOVA analysis, after correction of biofilm age factor (Table 1, RHG, Medium:Age, *F* = 1.1, *p* = 0.36).

**Table 1:** One-way PERMANOVA analysis.

Substratum	Parameter	Pseudo F	*P*-value
RHG	Medium	4.2	0.006
RHG	Age	14.1	0.001
RHG	*Medium:Age*	1.1	0.358
HAP	Medium	14.0	0.001
HAP	Age	7.6	0.001
HAP	*Medium:Age*	5.6	0.002
TI	Medium	45.5	0.001
TI	Age	5.1	0.002
TI	*Medium:Age*	3.7	0.003

The effect of medium type and biofilm age on microcosm biofilm composition grown on each type of substratum were analyzed with two-way PERMANOVA analysis, based on Bray-Curtis distance and permutation of 999.

Cladograms plotted from LEfSe analysis identified a few substratum-specific genera or higher level, irrespective of biofilm growth media (Fig. 3b). RHG-biofilms had a higher abundance of *Haemophilus* and *Porphyromonas*; HAP-biofilms had a higher abundance of *Bacilli* and TI biofilms had a higher abundance of *Prevotella* (Fig. 3c). For *Bacilli*, the differential plots of two genera, *Staphylococcus* and *Streptococcus*, are shown. These plots further supported the results observed from the cladograms.

### Microbial correlation within microcosm biofilms

**Fig. 4:**
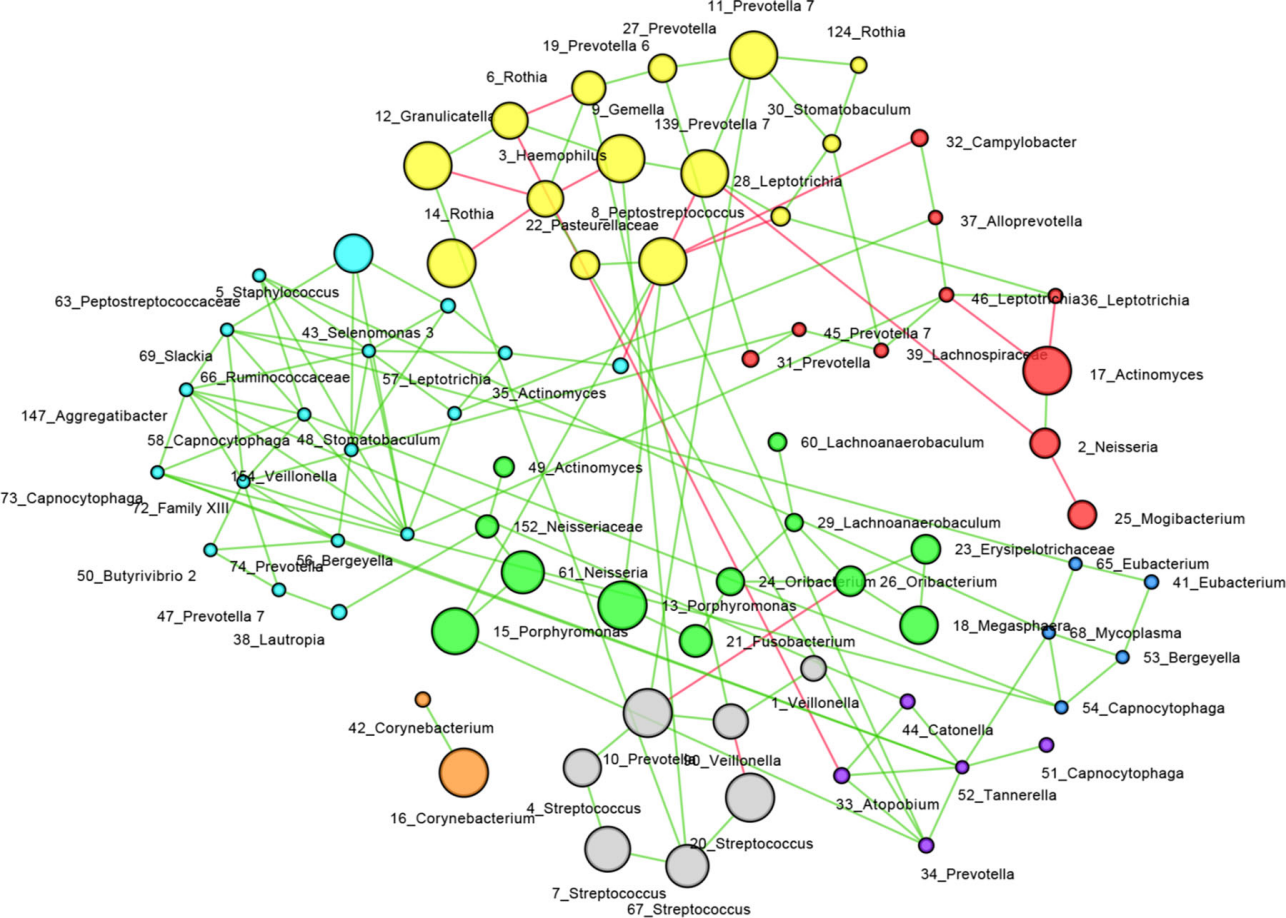
Microbial co-occurrence network. Microbial interactions were inferred with SPIEC-EASI algorithm using “MB” neighborhood selection procedure. Each node represents an OTU. The green edges represent positive correlation between taxa and the red ones represent negative correlation. The diameter of the node corresponds to the relative abundance of the OTU. Clusters in the network were identified using the Girvan-Newman clustering algorithm. The nodes in one cluster are labeled in the same color. The number in the name of each node represents its OTU number.

SPIEC-EASI was developed for the inference of microbial ecological networks from amplicon sequencing datasets^20^. Using the SPIEC-EASI algorithm, we are able to illustrate the overall microbial interaction within the different microcosm biofilms (Fig. 4). A total of 129 microbial interactions were identified, indicated by the number of edges between the nodes. Among these interactions, 15 were negative and 114 were positive. The average node degree was 3.5 (± 1.7). OTU_8, 56, 66 and 72 have the highest number of degrees. Using the Girvan-Newman clustering algorithm, a further 8 clusters were identified. Interestingly, the OTUs assigned as aerobic *Neisseria* and *Rothia* were clustered with those assigned as strictly-anaerobes *Porphyromonas* and *Prevotella*. There was a positive interaction between *Neisseria* and *Porphyromonas* but the interactions between *Rothia* and *Prevotella* could be both positive and negative.

## Discussion

Niche-specific community structures are important for the stability of microbial communities in the oral cavity and hence critical in maintaining healthy homeostasis^7,9^. It has been shown that site-specific adhesion, nutrient availability and substratum type might play important roles in modulating the niche-specificity of microbiota^9,11,12^. This study investigated the latter two factors in an in vitro biofilm model and standardized the adhesion by mixing the biofilm inoculum in agar and place the agar on top of the substratum. Our data revealed that nutrient availability (biofilm growth media) and substratum type influenced the microbiome structure. When the biofilms grew on the surface of the biotic substratum RHG, the microbial profiles were independent of the type of growth media. In contrast, when the biofilms grew on abiotic substrata, HAP and TI, the microbial profiles were considerably affected by the type of growth media. Moreover, different bacterial genera (or higher level) were differentially abundant in the biofilms on various substrata: *Haemophilus* and *Porphyromonas* for RHG; *Bacilli* for HAP and *Prevotella* for TI.

A previous study reported that the growth media (SHI medium versus a modified artificial saliva medium with cysteine), not the substrata (glass versus HAP), affected the microbiome structure of in vitro biofilms^12^. However, our results indicate an interactive effect of growth media and substratum type on the microbiome structure. When the medium was TP, our results were in line with Li et al.^12^, where the biofilm viability and composition were not affected by the substratum type. TP medium was developed to mimic the nutrients available in the subgingival environment^13^. It is a complex medium comparable to the biofilm media used in Li’s study, which might explain the similar findings. In contrast, when using BHI medium, the biofilms were affected by the types of abiotic substrata: viability of HAP-biofilm first reduced and then recovered, but viability of TI-biofilm continuously decreased resulting in different species becoming predominant. To understand the observed decreasing viability in TI-biofilm and reduced Shannon index in HAP-biofilm when grown in BHI, we measured the pH of all biofilms and found that the biofilm pH was neutral except the pH of TI-biofilm grown in BHI, which was around 5.5 throughout the entire experiment. Hence, it is possible that the low buffer capacity of BHI was not sufficient to neutralize the acid produced by the biofilms on the surfaces of TI, resulting in low viability of TI-biofilm. In the case of HAP-biofilm, the initial pH-drop possibly led to the dissolution of HAP which subsequently prevented the pH from dropping further. As a result, the initial pH drop led to reduced biofilm viability followed by a neutral pH favoring the growth of specific bacterial species, e.g., *Staphylococcus*. Taken together, our data indicated that the microbial composition was shaped by a combination of multiple factors, including nutrient availability, environmental pH and substratum type.

Here we investigated the composition of oral multi-species biofilms grown on the surface of a biological tissue. Surprisingly, BHI, without any additional supplements, did not lead to any loss in viability or low species richness in RHG-biofilm, which is in contrast to HAP-biofilm and TI-biofilm. Microbial viability and composition in BHI were the same as those in TP. The components in TP, such as mucins and proteose peptone, were believed to be crucial for supporting the cultivation of fastidious bacteria and increasing the proportion of anaerobes^13,16,21–23^. Although BHI is a nutrient-rich medium, it misses substances, like haemin and vitamin K, which are critical for the growth of black-pigmented bacterial species, e.g., *Porphyromonas gingivalis*^24^. BHI supported a highly diverse bacterial community in vitro only when supplemented with mucins, haemin and vitamin K^21^. In another study, even the supplemented BHI was reported to cause low viability and low species richness in biofilms^22^. Therefore, our results indicate that living RHG tissues are able to provide key nutrient components for the growth of various bacteria and supported a highly diverse microbiome. Furthermore, the neutral pH of RHG-biofilm grown in BHI indicated that RHG might either neutralize the pH directly or reduce the acid production indirectly by limiting the growth of acid producing bacteria. Histological analysis of RHG revealed a moderate disruption in RHG epithelium both in agar and microcosm + agar groups compared to unexposed RHG which was probably caused by exposing to the high temperature of unsolidified agar (around 50 °C) during inoculation. We are currently exploring an improved inoculation method in order to maintain an optimal epithelium after application of agar. Nevertheless, our data do convincingly demonstrate the sufficient nutrient support of RHG to the microcosm biofilms over time.

Another interesting finding was the positive interaction between aerobic *Neisseria* and strictly anaerobic *Porphyromonas* through our network analysis. Notably, all biofilms were grown in 7.5% CO_2_ with ambient air in this study. Surprisingly, the strictly-anaerobic bacteria, *Porphyromonas*, *Fusobacterium* and *Prevotella*, were detected in our microcosm biofilms. Lambooij et al.^25^ showed that *Candida albicans* was able to induce the growth of strictly-anaerobic oral bacteria in air through mitochondrial activity. In order to illustrate the interaction between aerobic and anaerobic bacteria, we performed an additional experiment, where the strictly-anaerobic *P. gingivalis* was cultured with or without aerobic *Neisseria perflava*. The results clearly showed that the full growth of *P. gingivalis* in 7.5% CO_2_ could be achieved only when *N. perflava* was present (Supplementary Fig. 2). This set of data further supported the findings based on network analysis and demonstrated the corporation among bacterial species.

To exclude the effect of microbial site-specific adhesion, we grew microcosm biofilms by mixing the saliva inoculum with 0.7% agarose. This gel-entrapped biofilm model allows microbes to form a dense and structured biofilm within the gel matrix and mimics wound or lung biofilms in vivo^26,27^. Interestingly, we found the composition of microcosm biofilms was rather similar to that of the saliva inoculum, with a high BC similarity index of 0.7–0.8. The reasons for this relatively high similarity are not clear. It is possible that exclusion of initial bacterial attachment phase in our model avoided losing some bacterial species which were initially present in the inoculum but did not attach to the substrata. Furthermore, Baraniya et al.^22^ pointed out that high serum concentration (10–20%) could reduce the composition similarity between biofilms and inocula and recommended 5% serum in biofilm growth medium. Our model showed that high similarity could be achieved even without serum addition to the growth medium.

In this study, all microcosm biofilms were grown from the saliva collected from one donor. A previous study^19^ showed that the biofilm growth condition (for example, growth medium) seemed to have greater influence on biofilm compositions than the source of saliva inoculum (from individual or multiple donors). However, another study found that the in vitro biofilm compositions varied among different saliva donors^17^. Further studies are needed to understand the donor effect on in vitro microcosm biofilms.

In conclusion, this in vitro study demonstrated that the type of media and substrata interactively influenced viability and composition of the microcosm biofilms. RHG, the biotic substratum, was able to support a highly viable and diverse microbiome, even in nutrient poor growth media which lacks some essential growth elements. In contrast, the viability and diversity of the biofilms on the abiotic substrata were influenced by the substrata type, pH of the environment and the richness of the growth media. These results suggest that the host (oral mucosa) plays a vital role in the oral ecology.

## Methods

### Substrata

RHG was constructed using immortalized human gingival keratinocyte (KC-TERT, OKG4/bmi1/TERT, Rheinwald Laboratory, Boston, MA, USA) and fibroblast (Fib-TERT, T0026, ABM, Richmond, BC, Canada) cell lines^28^. In detail, fibroblasts (8 × 10^5^ cells/ml) were mixed with a collagen hydrogel and transferred to transwell inserts of 0.4 μM pores (Corning, NY, USA) in a 6-well tissue culture plate. After 24 h, keratinocytes were seeded on top of the fibroblast-collagen hydrogel at a density of 5 × 10^5^ cells/well. After 3 days submerged, RHGs were then lifted to the air-liquid interface to induce epithelial differentiation and cultured for another 10 days in the RHG differentiation medium. The RHG differentiation medium contains DMEM/Ham’s F12 (Gibco, Grand Island, USA), supplemented with 1% Fetal Clone III (GE, Logan, UT, USA), 1% penicillin-streptomycin (Gibco), 0.1 μM insulin (Sigma-Aldrich, St. Louis, MO, USA), 2 μM hydrocortisone (Sigma-Aldrich), 1 μM isoproterenol (Sigma-Aldrich), 10 μM carnitine (Sigma-Aldrich), 10 mM l-serine (Sigma-Aldrich), 0.4 mM l-ascorbic acid (Sigma-Aldrich) and 2 ng/mL epidermal growth factor (Sigma-Aldrich). The medium was changed every 3 days, and the RHGs were grown at 37 °C, 7.5% CO_2_. One day before the addition of microcosm biofilms, the RHG was refreshed with the same medium but without penicillin-streptomycin and hydrocortisone. During microcosm biofilm exposure, RHG, cultured in 6-well plates, were refreshed once daily with the RHG differentiation medium without penicillin-streptomycin and hydrocortisone.

Standardized HAP discs (diameter: 9.5 mm, thickness: 2 mm, HIMED) and TI discs (diameter: 10 mm, thickness: 1 mm, Baoji Titanium Industry) without any surface treatment were sterilized by autoclaving before use.

### Saliva inoculum

Unstimulated saliva was collected from a single healthy donor who was systemically healthy, had no periodontal disease or active caries, and had not taken any antibiotics for at least 3 months. The study was approved by the Medical Ethical Committee of the University Medical Center, Amsterdam UMC (document number 2011/236) with signed and informed consent from the donor. On the day of saliva collection, the donor was asked not to brush the teeth for 24 h and not to drink or eat for at least 2 h prior to sampling. The collected saliva was mixed with 60% glycerol (ratio of 1:1) and stored at −80 °C before use.

### Microcosm biofilm formation

The microcosm biofilms were grown in 0.7% agar (BD Biosciences)^29^ containing biofilm growth medium, BHI or TP, on 3 substrata. The Brain heart infusion broth (BHI) was prepared following the instruction of the manufactory (BD Difco). The Thompson medium (TP) contains 0.25% pig gastric mucin, 0.07% Bacto proteose peptone (BD Biosciences), 0.1 mol/L K_2_HPO_4_, 0.3% trypticase peptone (BBL, 211921), 0.5% yeast extract, 0.25% KCl, 10 μg/ml hemin, 1 μg/ml menadione, 0.05% cysteine hydrochloride, 1 mmol/L lysine, 1 mmol/L glycine, 1 mmol/L urea and 5 mmol/L arginine^13^. In contrast to original TP, the TP in this study does not contain fetal horse serum.

To form microcosm biofilms, a saliva stock was diluted in a freshly-prepared mixture of BHI or TP with 0.7% agar to reach 10^7^ CFU of total viable cells per sample and inoculated onto the surface of each substratum (RHG: 50 μL, HAP disc: 100 μL, TI disc: 100 μL). The reduced volume on air-exposed RHG was used to prevent contamination of culture medium from the overflow of the inoculum during co-culture. For RHG, a control of 50 μL BHI-agar or TP-agar without saliva inoculum was included as well as an unexposed RHG. At inoculation, the sterile HAP and TI discs were placed in 24-well plates (one disc/well). The 6-well plates containing RHG with biofilms and the 24-well plate containing HAP or TI discs with biofilms were incubated at 37 °C, 7.5% CO_2_.

On day 1, 3 and 5 after inoculation, biofilm samples were harvested for viable cell counts and microbial composition analysis. The RHGs were fixed in 4% formaldehyde and processed for paraffin embedment. Tissue sections (5 μm) were stained with hematoxylin and eosin (H&E).

All experiments were repeated 3 times and duplicate samples was included in each experiment.

### Viable cell counts of microcosm biofilms

Each biofilm sample was collected using a microbrush into 2 mL cysteine peptone water (CPW). The biofilms were dispersed by 10 s vortexing, followed by 1 min sonication on ice 1 s (with 1 s pulse) at an amplitude of 40 W (Vibra cell, Sonics & Materials). Each sample was then serially diluted and plated onto trypticase soy agar containing 5% sheep blood, 5 μg/mL hemin and 1 μg/mL menadione, and further incubated anaerobically at 37 °C for 7 days before the colony forming units (CFUs) were counted. The remaining sonicated samples were centrifuged and stored at −80 °C for sequencing.

### 16S rDNA sequencing and microbial compositional analysis

Genomic DNA of the biofilm pellets was extracted and the V4 region of 16 S rDNA was amplified according to established protocols^30,31^. After purification, paired-end sequencing of the amplicons (251-bp length) was conducted on the Illumina MiSeq platform at the UMC Cancer Center Amsterdam (Amsterdam, the Netherlands) using the MiSeq reagent kit V3 (Illumina, San Diego, CA, USA). The obtained paired-end reads were quality-filtered, merged and clustered into operational taxonomic units (OTUs) at 97% similarity. The most abundant sequence of each OTU was assigned a taxonomy using the ribosomal database project (RDP) classifier (min. confidence 0.8) and the SILVA rRNA database (v132)^32^. The OTU table was subsampled at a depth of 7900 reads per sample.

### Data analysis and statistics

Viable counts were log10 transformed and analyzed for the changes in time for each substratum using one-way ANOVA in SPSS (version 25). The viable counts of day 1 were also compared to those of the inoculum using Student’s *t* test. Differences were considered statistically significant if *p* < 0.05.

Several analyses on the OTU table were conducted in PAST (paleontological Statistics version 4.03)^33^: namely Shannon diversity index, principal component analysis (PCA) and one-way permutational multivariate analysis of variance (PERMANOVA). The OTU table was log2-transformed before the latter two analyses.

Linear discriminant analysis effect size (LEfSe)^34^ and network analysis were conducted on the OTUs with a relative abundance higher than 0.01%. To perform a LEfSe analysis, the relative abundance data were first split by biofilm growth media into two groups, BHI and TP. Next, LEfSe was carried out for each medium separately, using the type of substrata as class with default settings, except that the threshold on the logarithmic LDA score for discriminative features was set to 4.0. The interaction between bacterial species in the biofilms was inferred using Sparse Inverse Covariance Estimation for Ecological Association and Statistical Inference (SPIEC-EASI, v1.0.7)^20^. The parameters of SPIEC-EASI are: “MB” neighborhood selection procedure, lambda minimum ratio of 1 × 10^−2^, nlambda of 20 and 50. The SPIEC-EASI calculated weighted network adjacency network was generated in R (v4.0.3) and imported into Cytoscape (v3.8.2). A community clustering based on Girvan-Newman fast greedy algorithm in clusterMaker plugin was used to identify the clusters inside the network. The identified clusters were visualized in Cytoscape using different color labels.

### Reporting summary

Further information on research design is available in the Nature Research Reporting Summary linked to this article.

## Supplementary information

Reporting Summary

Supplementary Information

## Data Availability

The data that support the findings of this study are available from the corresponding author upon reasonable request. The sequencing data have been submitted to the NCBI BioProject database under accession number PRJNA754106.

## References

[1] Human Microbiome Project, C. (2012). Structure, function and diversity of the healthy human microbiome. Nature.

[2] Xu X (2015). Oral cavity contains distinct niches with dynamic microbial communities. Environ. Microbiol.

[3] Dong L (2018). Microbial similarity and preference for specific sites in healthy oral cavity and esophagus. Front Microbiol.

[4] Ursell LK, Metcalf JL, Parfrey LW, Knight R (2012). Defining the human microbiome. Nutr. Rev..

[5] Oh J (2013). The altered landscape of the human skin microbiome in patients with primary immunodeficiencies. Genome Res.

[6] Pletcher SD, Goldberg AN, Cope EK (2019). Loss of microbial niche specificity between the upper and lower airways in patients with cystic fibrosis. Laryngoscope.

[7] Lamont RJ, Koo H, Hajishengallis G (2018). The oral microbiota: dynamic communities and host interactions. Nat. Rev. Microbiol.

[8] Aas JA, Paster BJ, Stokes LN, Olsen I, Dewhirst FE (2005). Defining the normal bacterial flora of the oral cavity. J. Clin. Microbiol.

[9] Mark Welch JL, Dewhirst FE, Borisy GG (2019). Biogeography of the oral microbiome: the site-specialist hypothesis. Annu. Rev. Microbiol.

[10] Mark Welch JL, Rossetti BJ, Rieken CW, Dewhirst FE, Borisy GG (2016). Biogeography of a human oral microbiome at the micron scale. Proc. Natl Acad. Sci. USA.

[11] Daubert D, Pozhitkov A, McLean J, Kotsakis G (2018). Titanium as a modifier of the peri-implant microbiome structure. Clin. Implant Dent. Relat. Res.

[12] Li, B. et al. Effects of different substrates/growth media on microbial community of saliva-derived biofilm. *FEMS Microbiol. Lett.* **364**, 10.1093/femsle/fnx123 (2017). 10.1093/femsle/fnx123 28854684

[13] Thompson H, Rybalka A, Moazzez R, Dewhirst FE, Wade WG (2015). In vitro culture of previously uncultured oral bacterial phylotypes. Appl Environ. Microbiol.

[14] Peyyala R, Kirakodu SS, Novak KF, Ebersole JL (2013). Oral epithelial cell responses to multispecies microbial biofilms. J. Dent. Res..

[15] Kommerein N (2017). An oral multispecies biofilm model for high content screening applications. PLoS ONE.

[16] Fernandez YMM (2017). A reproducible microcosm biofilm model of subgingival microbial communities. J. Periodontal. Res..

[17] Cieplik F (2019). Microcosm biofilms cultured from different oral niches in periodontitis patients. J. Oral. Microbiol..

[18] Shang L (2018). Multi-species oral biofilm promotes reconstructed human gingiva epithelial barrier function. Sci. Rep..

[19] Buskermolen JK, Janus MM, Roffel S, Krom BP, Gibbs S (2018). Saliva-derived commensal and pathogenic biofilms in a human gingiva model. J. Dent. Res..

[20] Kurtz ZD (2015). Sparse and compositionally robust inference of microbial ecological networks. PLoS Comput. Biol..

[21] Kistler JO, Pesaro M, Wade WG (2015). Development and pyrosequencing analysis of an in-vitro oral biofilm model. BMC Microbiol..

[22] Baraniya D (2020). Modeling normal and dysbiotic subgingival microbiomes: effect of nutrients. J. Dent. Res..

[23] Ramage G (2017). The epithelial cell response to health and disease associated oral biofilm models. J. Periodontal Res..

[24] Mayrand D, Holt SC (1988). Biology of asaccharolytic black-pigmented Bacteroides species. Microbiol Rev..

[25] Lambooij JM, Hoogenkamp MA, Brandt BW, Janus MM, Krom BP (2017). Fungal mitochondrial oxygen consumption induces the growth of strict anaerobic bacteria. Fungal Genet. Biol..

[26] Crone S, Garde C, Bjarnsholt T, Alhede M (2015). A novel in vitro wound biofilm model used to evaluate low-frequency ultrasonic-assisted wound debridement. J. Wound Care.

[27] Pabst B, Pitts B, Lauchnor E, Stewart PS (2016). Gel-entrapped Staphylococcus aureus bacteria as models of biofilm infection exhibit growth in dense aggregates, oxygen limitation, antibiotic tolerance, and heterogeneous gene expression. Antimicrob. Agents Chemother..

[28] Buskermolen JK (2016). Development of a full-thickness human gingiva equivalent constructed from immortalized keratinocytes and fibroblasts. Tissue Eng. Part C. Methods.

[29] Strathmann M, Griebe T, Flemming HC (2000). Artificial biofilm model—a useful tool for biofilm research. Appl. Microbiol. Biotechnol..

[30] Koopman JE (2016). Nitrate and the origin of saliva influence composition and short chain fatty acid production of oral microcosms. Micro. Ecol..

[31] Han Q (2019). Regrowth of microcosm biofilms on titanium surfaces after various antimicrobial treatments. Front. Microbiol..

[32] Quast C (2013). The SILVA ribosomal RNA gene database project: improved data processing and web-based tools. Nucleic Acids Res..

[33] Hammer Ø, Harper D, Ryan P (2001). PAST-palaeontological statistics, ver. 1.89. Palaeontol. Electron.

[34] Segata N (2011). Metagenomic biomarker discovery and explanation. Genome Biol..

