# Supplementary material for: Saliva-derived microcosm biofilms grown on different oral surfaces in vitro
Source: NPJ Biofilms Microbiomes. 2021 Sep 9;7:74. doi: 10.1038/s41522-021-00246-z (PMC8429667; doi:10.1038/s41522-021-00246-z)
Supplement: Supplementary file 1 — Reporting Summary [file 41522_2021_246_MOESM1_ESM.pdf]

nature portfolio

Corresponding author(s): Dongmei Deng

Last updated by author(s): Aug 2, 2021

Reporting Summary

Nature Portfolio wishes to improve the reproducibility of the work that we publish. This form provides structure for consistency and transparency in reporting. For further information on Nature Portfolio policies, see our Editorial Policies and the Editorial Policy Checklist.

Statistics

For all statistical analyses, confirm that the following items are present in the figure legend, table legend, main text, or Methods section.

n/a	Confirmed	
☐	☒	The exact sample size (n) for each experimental group/condition, given as a discrete number and unit of measurement
☐	☒	A statement on whether measurements were taken from distinct samples or whether the same sample was measured repeatedly
☒	☐	The statistical test(s) used AND whether they are one- or two-sided<br>*Only common tests should be described solely by name; describe more complex techniques in the Methods section.*
☒	☐	A description of all covariates tested
☐	☒	A description of any assumptions or corrections, such as tests of normality and adjustment for multiple comparisons
☐	☒	A full description of the statistical parameters including central tendency (e.g. means) or other basic estimates (e.g. regression coefficient) AND variation (e.g. standard deviation) or associated estimates of uncertainty (e.g. confidence intervals)
☐	☒	For null hypothesis testing, the test statistic (e.g. F, t, r) with confidence intervals, effect sizes, degrees of freedom and P value noted<br>*Give P values as exact values whenever suitable.*
☒	☐	For Bayesian analysis, information on the choice of priors and Markov chain Monte Carlo settings
☐	☒	For hierarchical and complex designs, identification of the appropriate level for tests and full reporting of outcomes
☒	☐	Estimates of effect sizes (e.g. Cohen's d, Pearson's r), indicating how they were calculated

Our web collection on statistics for biologists contains articles on many of the points above.

Software and code

Policy information about availability of computer code

Data collection	Data has been processed using standard methods available online, namely USEARCH and QIIME (https://www.drive5.com/usearch/, http://qiime.org/). Details on how this software was used are available in the manuscript and the cited publication therein (Koopman et al, 2016 doi 10.1007/s00248-016-0775-z). In short, UPARSE pipeline using USEARCH v8.0.1623 for merging paired-end reads and clustering the sequences into OTUs (with the following adaptations: cluster_otus with -uparse_maxdball 1200, , and usearch_global with -maxaccepts 8 -maxrejects 64 -maxhits 1). QIIME v1.8.0, using the RDP classifier and SLIVA rRNA database (v132) for assigning taxonomy.
Data analysis	SPSS (version 25); Prism 8 (version 8.2.1); PAST (version 4.03); LEfse (online Galaxy version 2.0); SPIEC-EASI (version 1.0.7); Cytoscape (version 3.8.2)

For manuscripts utilizing custom algorithms or software that are central to the research but not yet described in published literature, software must be made available to editors and reviewers. We strongly encourage code deposition in a community repository (e.g. GitHub). See the Nature Portfolio guidelines for submitting code & software for further information.

Data

Policy information about availability of data

All manuscripts must include a data availability statement. This statement should provide the following information, where applicable:

- Accession codes, unique identifiers, or web links for publicly available datasets
- A description of any restrictions on data availability
- For clinical datasets or third party data, please ensure that the statement adheres to our policy

The data that support the findings of this study are available from the corresponding author upon reasonable request. The sequencing data have been submitted to the NCBI BioProject database under accession number PRJNA754106.

Field-specific reporting

Please select the one below that is the best fit for your research. If you are not sure, read the appropriate sections before making your selection.

☒ Life sciences ☐ Behavioural & social sciences ☐ Ecological, evolutionary & environmental sciences

For a reference copy of the document with all sections, see nature.com/documents/nr-reporting-summary-flat.pdf

Life sciences study design

All studies must disclose on these points even when the disclosure is negative.

Sample size	The sample size per group was determined based on the previous biofilm studies. All experiments were repeated 3 times. Each experiment included duplicate samples per group.
Data exclusions	The data was excluded for sequencing analysis due to the following reasons: no sequencing data (DNA yield is too low) or the reads are lower than 7900 (the depth of subsampling). In total, 4 samples were lost.
Replication	All experiments were repeated 3 times. Each experiment included duplicate samples per group. The results were reproducible.
Randomization	This is not relevant for this study, because this is an in vitro study.
Blinding	The blinding is not relevant for this study, because the data obtained were measurements. The data cannot be influenced by the researchers.

Reporting for specific materials, systems and methods

We require information from authors about some types of materials, experimental systems and methods used in many studies. Here, indicate whether each material, system or method listed is relevant to your study. If you are not sure if a list item applies to your research, read the appropriate section before selecting a response.

Materials & experimental systems

n/a	Involved in the study	
☒	☐	Antibodies
☒	☐	Eukaryotic cell lines
☒	☐	Palaeontology and archaeology
☒	☐	Animals and other organisms
☐	☒	Human research participants
☒	☐	Clinical data
☒	☐	Dual use research of concern

Methods

n/a	Involved in the study	
☒	☐	ChIP-seq
☒	☐	Flow cytometry
☒	☐	MRI-based neuroimaging

Human research participants

Policy information about studies involving human research participants

Population characteristics	One healthy saliva donor. The age and gender of the donor is irrelevant for this study.
Recruitment	The donor is systematically healthy, has no periodontal disease or active caries and has not taken any antibiotics for at least 3 months. This information is based on the self-report of the donor.
Ethics oversight	The study was approved by the Medical Ethical Committee of the VU University Medical Center Amsterdam (document number 2011/236)

Note that full information on the approval of the study protocol must also be provided in the manuscript.
