# Supplementary material for: Saliva-derived microcosm biofilms grown on different oral surfaces in vitro
Source: NPJ Biofilms Microbiomes. 2021 Sep 9;7:74. doi: 10.1038/s41522-021-00246-z (PMC8429667; doi:10.1038/s41522-021-00246-z)
Supplement: Supplementary file 2 — Supplementary Information [file 41522_2021_246_MOESM2_ESM.pdf]

## Supplementary Figure 1

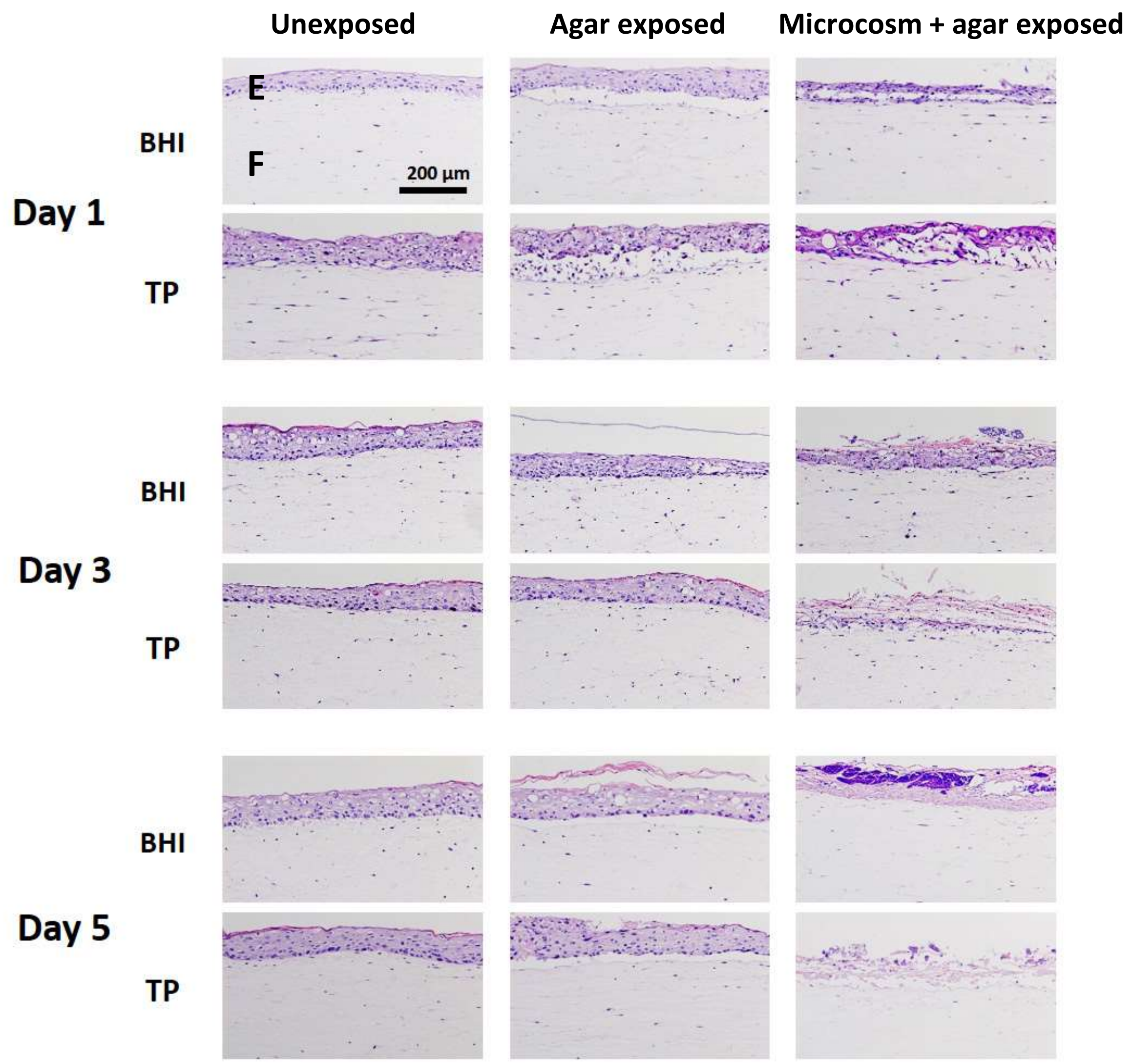


**Histology of RHG under different exposure conditions for 1, 3 or 5 days.**

Haematoxylin and eosin staining of 5 µm paraffin embedded tissue sections. Unexposed: RHG without exposure. Agar exposed: RHG exposed to 0.7% agar containing growth media (either BHI or TP). Microcosm + agar exposed: RHG exposed to 0.7% agar containing microcosm biofilm and one of the growth media (either BHI or TP). E, gingival keratinocytes formed stratified epithelium. F, gingival fibroblast-populated hydrogel. The growth media are BHI and Thompson (TP). Data represent three independent experiments with intra-experimental duplicates.

The images in agar exposed and microcosm + agar exposed groups showed that in certain locations epithelium came loose from hydrogel during tissue processing and embedding and possibly due to agar application.

## Supplementary Figure 2

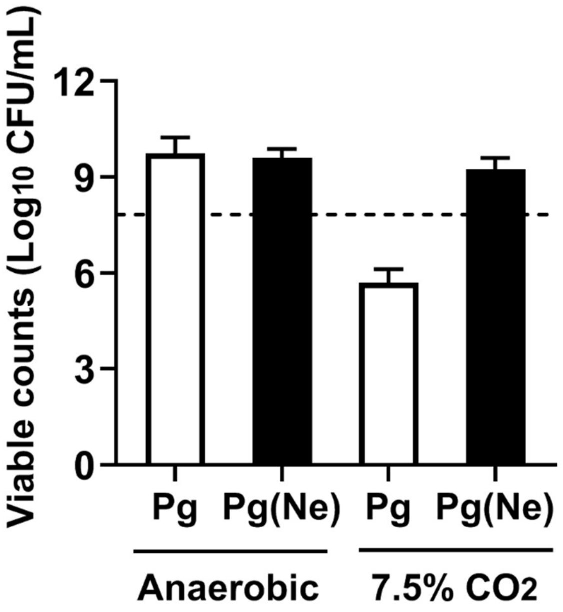


**The viable cell counts of *P. gingivalis* with or without *N. perflava***

Pg: *Porphyromonas gingivalis* ATCC33277 only. Pg(Ne): *P. gingivalis* co-cultured with *Neisseria perflava* ATCC14799. The dash line indicates the initial level of viable counts of *P. gingivalis*. The experiment was performed twice. Data are shown as mean ± SD.

To investigate the interaction between aerobic and anaerobic bacteria, we grew individual *P. gingivalis*, *N. perflava* and the co-culture of these two bacteria at 7.5% $CO_2$ (with ambient air) or anaerobically. Briefly, the 24-h pre-cultures of *P. gingivalis* and *N. perflava* were first adjusted to an optical density at 600 ($OD_{600}$) of 1. The OD 1 culture of *P. gingivalis* was then diluted 50 times in the fresh growth medium (BHI supplemented with 10 µg/mL hemin and 1 µg/mL menadione) alone or with the addition of *N. perflava* culture. The final cell counts in the dilutions were 7.8 logCFU/ml for *P. gingivalis* and/or 6.7 logCFU/ml for *N. perflava*. The dilutions were incubated for 24 h either at 7.5% $CO_2$ or anaerobically (10% $CO_2$, 10% $H_2$ and 80% $N_2$) at 37°C. The viable cell counts were assessed by

plating on trypticase soy agar containing 5% sheep blood, 5 µg/mL hemin and 1 µg/mL menadione. The plates were incubated anaerobically for 7 days before counting the CFUs of *P. gingivalis.*

The supplementary figure 2 shows that under anaerobic culture conditions, the viable counts of *P. gingivalis* increased 2 logs within 24 hours, irrespective of the absence or presence of *N. perflava*. However, under aerobic conditions (7.5% $CO_2$), the 2-log increase of the viable counts was only observed when *N. perflava* was present. The viable counts of the monoculture dropped 2 logs.
